# Population-based analysis of curative therapies in stage II non-small cell lung cancer: the role of radiotherapy in medically inoperable patients

**DOI:** 10.1186/s13014-020-1466-y

**Published:** 2020-01-30

**Authors:** Sara Moore, Bonnie Leung, Jonn Wu, Cheryl Ho

**Affiliations:** 1 University of British Columbia, Vancouver, BC V6T 1Z4 Canada; 2 Department of Medical Oncology, BC Cancer 600 W 10th Avenue Vancouver, Vancouver, BC V5Z 4E6 Canada; 3 Department of Radiation Oncology, BC Cancer 600 W 10th Avenue Vancouver, Vancouver, BC V5Z 4E6 Canada

**Keywords:** Non-small cell lung cancer, Stage II, Inoperable, Real-world, Outcomes

## Abstract

**Objectives:**

Curative intent therapy of stage II NSCLC may include surgical resection or definitive radiotherapy. Primary management with surgery or radiotherapy may be influenced by patient and disease characteristics. We sought to perform a comparison of patients receiving surgery or radical radiation therapy as their curative treatment, and explore the impact of known prognostic factors on outcome.

**Materials and methods:**

A retrospective review was completed of all patients with stage II NSCLC referred to the BC Cancer Agency from 2005 to 2012. Cases were filtered to identify those receiving curative intent therapy including surgery or radiotherapy. Information was collected on known prognostic and predictive factors. The primary outcome measure was overall survival. We compared survival among patients receiving curative intent radiotherapy versus surgical intervention.

**Results:**

A total of 535 patients were referred. Of these, 245 (46%) received curative intent surgery, 132 (25%) curative intent radiotherapy, and 158 (30%) did not receive curative therapy. There were significant differences between cohorts with respect to median age, histology, ECOG PS, smoking status, and weight loss. Median OS was significantly different between cohorts: 61.4 m surgery, 26.5 m curative RT, and 13.1 m non-curative therapy. In a case-matched analysis, median OS remained superior for surgery at 101.6 m vs 28.1 m for curative RT. In a multivariate analysis, ECOG PS, weight loss, and treatment cohort all influenced survival. Among patients receiving curative intent radiotherapy, the use of concurrent chemotherapy and RT dose > = 60Gy were associated with improved outcomes.

**Conclusions:**

Among patients with stage II NSCLC, many are unable to undergo standard of care surgical resection. Radiotherapy provides an inferior yet still curative option in the management of inoperable patients. Further work is needed to optimize outcomes in this population.

## Background

Non-small cell lung cancer (NSCLC) is the most frequently diagnosed cancer worldwide [1]. Approximately one-third of patients will present with early stage disease [2], and with recent evidence to support lung cancer screening, it is expected this will become a growing patient population [3, 4]. In the American Joint Committee on Cancer (AJCC) 8th edition, stage II NSCLC encompasses patients with T2b/3 N0 and T1/2N1 disease [5]. Despite being amenable to potentially curative therapy, the survival outcomes in stage II NSCLC are mediocre, with a 5 year overall survival (OS) of 53–60% with clinical staging and 56–65% with pathologic staging [5].

The standard of care for stage II NSCLC is surgery [6, 7], followed by adjuvant chemotherapy in higher risk patients [8–11]. There are a proportion of patients who are deemed medically inoperable due to comorbidities or refuse surgery [12–16]. For inoperable stage II patients, the optimal treatment is much less certain. Chemoradiation or radical doses of radiotherapy alone remain a curative option, however there is limited evidence to guide this approach, and outcomes are unclear [17–19].

In this study, we examine the landscape of treatment of stage II NSCLC in a real-world population. We sought to explore outcomes in patients treated with curative intent radiotherapy compared to surgery, and clarify the role of radiotherapy in the medically inoperable population.

## Methods

### Population

A retrospective cohort study was performed of all patients with stage II (by AJCC 7th edition) NSCLC who were referred to BC Cancer between January 2005 and December 2012. BC Cancer is a provincial cancer program which serves a population of 4.6 million. Approximately 80% of patients with lung cancer in the province of British Columbia (BC) are referred to BC Cancer.

Three cohorts were created based on the type of therapy received:

- Curative intent surgery: sub-lobar resection, lobectomy, pneumonectomy +/− adjuvant chemotherapy
- Curative intent radiation (CurRT): Stereotactic body radiation therapy (SBRT) - 48Gy/4fr, 60Gy/8fr, 60Gy/15fr. Standard external beam radiotherapy - 50Gy/20fr, 50Gy/25fr, 55Gy/20fr, 60Gy/30fr, 66Gy/33fr +/− concurrent/sequential chemotherapy
- Non-curative therapy: systemic therapy, radiotherapy with palliative intent, or best supportive care

Patients who had another malignancy diagnosed within 5 years prior to lung cancer diagnosis, with the exception of non-melanomatous skin cancer or cervical cancer in situ, were excluded.

### Data collection

Data collection was facilitated by the Outcomes and Surveillance Integration System (OaSIS), a database that houses the Lung Tumor Group outcomes unit. Information was collected through OaSIS on known prognostic factors including: age, sex, smoking history, histology, Eastern Cooperative Oncology Group (ECOG) performance status (PS), and weight loss. Treatment details including chemotherapy, surgery, and radiotherapy were collected retrospectively.

### Statistical analysis

Statistical analysis was conducted using SPSS Version 23. Known prognostic factors were compared between the two cohorts using the Chi-Square test or Fisher’s Exact test where appropriate (categorical variables), and the Mann-Whitney test (continuous variables).

The primary outcome measure was overall survival (OS) calculated from the date of diagnosis. Survival curves were compared between cohorts using a log-rank test. A *p*-value of less than or equal to 0.05 was considered statistically significant. Multivariate analysis of potential factors associated with overall survival was performed using the Cox proportional hazards model.

Disease-free survival (DFS) was defined as the date of diagnosis to any recurrence (both local and distant) or death. DFS was compared between patients receiving curative intent surgery or radiotherapy. Recurrence was determined based on imaging and follow-up per treating physician discretion. Patients were censored by date of last follow-up visit at BC Cancer.

To account for the expected imbalance in prognostic factors between treatment cohorts, a case-matched OS analysis was performed. Patients who received surgery were matched 1:1 with patients receiving curative intent radiotherapy based on age (+/− 5 years), sex, ECOG (0–1 or 2), and smoking status (never, former, current).

An exploratory analysis was performed evaluating the potential effect of radiotherapy dose and the use of chemoradiation on survival. Patients receiving concurrent or sequential chemotherapy were compared to those treated with curative intent RT alone. Patients receiving curative RT were separated into 2 cohorts (> = 60Gy, 50-59Gy), and were compared to patients receiving moderate-high dose palliative radiotherapy (30-49Gy). Due to small numbers (*n* = 10), patients receiving SBRT were excluded from this analysis.

## Results

### Baseline patient characteristics

A total of 535 patients with stage II NSCLC were referred to BC Cancer during the study period. Of these, 245 (46%) received curative intent surgery, 132 (25%) curative intent radiotherapy, and 158 (30%) did not receive curative therapy. Baseline characteristics are presented in Table 1. In the entire population: median age 72, 51% female, 36% adenocarcinoma, 60% ECOG 0–1, 6% never smokers, and 52% weight loss < 5% body weight. There were significant differences between cohorts with respect to median age, histology, ECOG PS, smoking status, and weight loss.

**Table 1:** Baseline characteristics of surgery and radiation cohorts

		Surgery *n* = 245	Curative RT *n* = 132	Non-curative treatment *n* = 158	*p*-value	*p*-value Surg vs RT
Age	Median	67	73	78	< 0.001	< 0.001
	(range)	43–86	35–94	55–93		
Sex	Female	133 54%	61 46%	80 51%	0.322	0.135
	Male	112 46%	71 54%	78 49%		
Histology	Adenocarcinoma	138 56%	34 26%	20 13%	< 0.001	< 0.001
	Squamous	76 31%	42 32%	56 35%		
	NOS/other	31 13%	56 42%	82 52%		
ECOG	0–1	173 71%	87 66%	59 37%	< 0.001	0.614
performance	≥2	61 25%	39 30%	94 60%		
status	Unknown	11 5%	6 5%	5 3%		
Weight loss	< 5% body weight 5–10%	145 59% 39 16%	73 55% 16 12%	61 39% 25 16%	0.001	0.122
	> 10%	20 8%	21 16%	28 18%		
	Unknown	41 17%	22 17%	44 28%		
Smoking	Never	20 8%	3 2%	11 7%	0.046	0.053
	Former	113 46%	74 56%	74 47%		
	Current	109 45%	52 39%	65 41%		
	Unknown	3 1%	3 2%	8 5%		

Values are presented as n (%) unless otherwise indicated

*RT* radiation, *ECOG* Eastern Cooperative Oncology Group

Never smokers: less than 100 cigarettes over lifespan; former smokers: quit greater than 1 year ago; current smokers: actively smoking or quit less than 1 year ago

### Treatment

Details of therapy employed for patients receiving curative and palliative intent treatment are presented in Table 2. There was a significant difference in the use of chemotherapy in the surgery and radiotherapy cohorts (54% vs 26%, *p* < 0.001). Among patients receiving non-curative therapy, 109 (69%) received palliative radiotherapy.

**Table 2:** Therapy received

Surgery (*n* = 245)		
Surgical procedure	Sub-lobar resection	9 4%
	Lobectomy	204 83%
	Pneumonectomy	32 13%
Chemotherapy	None	112 46%
	Adjuvant	126 51%
	Neoadjuvant	7 3%
Curative Radiotherapy (n = 132)		
RT dose	≥66Gy	16 12%
	60-66Gy	43 33%
	SBRT	10 8%
	50-59Gy	63 48%
Chemotherapy	None	98 74%
	Concurrent	31 24%
	Sequential	3 2%
Non-curative treatment (*n* = 158)		
Palliative radiotherapy		109 69%
Systemic treatment*		12 8%
Best supportive care		44 28%

Values are presented as n (%)

*RT* radiation, *SBRT* stereotactic body radiation therapy

*7 patients received both palliative RT and systemic therapy

### Overall and disease-free survival

**Fig. 1:**
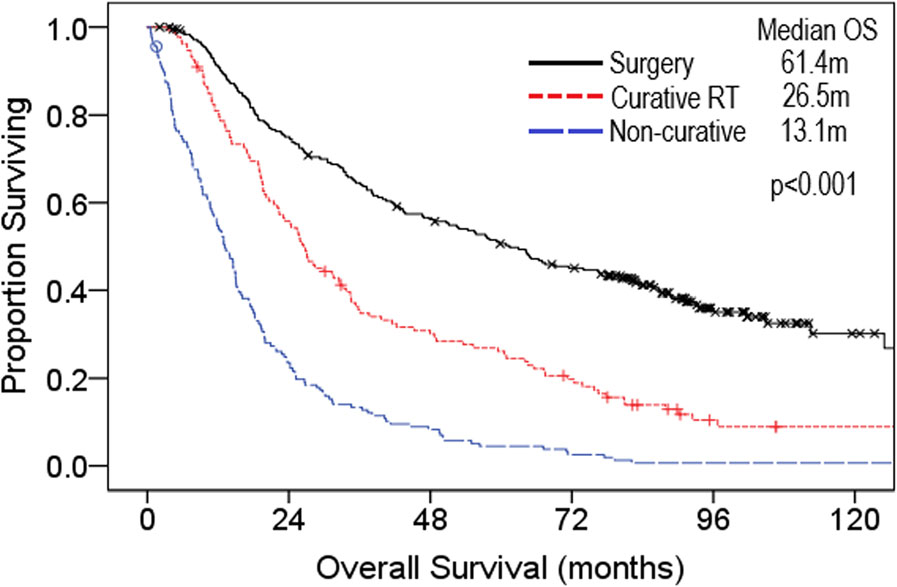
Kaplan-Meier overall survival curves for surgery (*n* = 245), curative radiation (*n* = 132), and non-curative treatment (*n* = 158) cohorts m, months; RT, radiotherapy

At the time of analysis, 426 (80%) of patients had died. Median OS was 27.1 m (months) in the entire population. Median OS was significantly different between cohorts: 61.4 m surgery, 26.5 m CurRT, and 13.1 m non-curative therapy (Fig. 1a). Estimated 5-year survival rates were higher in the surgery cohort at 50.6% (95% confidence interval [CI] 44.2–57.0%) compared to the CurRT cohort at 26.1% (95% CI 18.3–33.9%). Five-year OS was inferior in the cohort receiving non-curative therapy at 4.5% (95% CI 1.3–7.7%).

A total of 293 (78%) of patients receiving curative intent therapy developed recurrent disease or died. Median DFS was 27.1 m in the overall population. There was improved DFS with surgery with median DFS 36.4 m for surgery and 19.6 m for CurRT. Estimated 5-year DFS was 41.3% (95% CI 34.9–47.7%) for surgery and 15.9% (95% CI 9.3–22.5%) for CurRT.

In the surgery cohort 172 (70%) of patients had recurrent disease versus 121 (92%) in the radiotherapy cohort. There was a significant difference in the type of recurrence event: in the surgery cohort there was a higher proportion of local only recurrence (27% vs 19%), distant recurrence (46% vs 36%), and second primary (5% vs 1%) (*p* < 0.001). In the radiotherapy cohort, more patients died without a prior documented recurrence event (52% vs 20%). In the patients with recurrence, only a small fraction received subsequent systemic therapy: 27% the surgical and 19% in the radiotherapy cohort respectively.

### Univariate and multivariate analysis for OS

In patients receiving curative intent therapy, younger age, ECOG PS 0–1, adenocarcinoma histology, lack of weight loss, and never smoking were associated with improved overall survival in univariate analysis (Table 3). Comparing treatment cohorts, curative therapy with radiation was inferior to surgery (HR 2.06). Age, sex, histology, ECOG PS, weight loss, smoking status, and treatment cohort were included in a multivariate model. In this model, the impact of ECOG PS, weight loss, and treatment cohort remained significant.

**Table 3:** Univariate analysis and multivariate model of factors associated with overall survival in patients receiving curative intent therapy. HR > 1.0 indicates increased risk of death

		UVA		MVA		
		HR	*p*-value	HR	95% CI	*p*-value
Group	Surgery	Ref	< 0.001	Ref		
	Radiation	2.062		1.730	1.284–2.330	< 0.001
Age		1.038	< 0.001	1.013	0.998–1.029	0.094
Sex	Female	Ref	0.354	Ref		
	Male	1.094		0.979	0.757–1.267	0.873
Histology	Adenocarcinoma	Ref		Ref		
	Squamous	1.571	< 0.001	1.041	0.761–1.423	0.802
	NOS/other	1.997	< 0.001	0.955	0.679–1.342	0.791
ECOG	0–1	Ref	< 0.001	Ref		
	> = 2	2.156		1.590	1.210–2.090	0.001
Weight loss	< 5%	Ref		Ref		
	5–10%	1.100	0.510	0.847	0.585–1.226	0.378
	> 10%	2.294	< 0.001	1.668	1.139–2.444	0.009
	unk	1.105	0.438	0.783	0.539–1.136	0.197
Smoking	Never	Ref		Ref		
	Former	1.391	0.126	1.097	0.595–2.020	0.767
	Current	1.398	0.124	1.253	0.667–2.354	0.484
Chemotherapy	No	Ref	< 0.001	Ref		
	Yes	0.560		0.707	0.524–0.954	0.023

*UVA* univariate analysis, *MVA* multivariate analysis, *HR* hazard ratio, *CI* confidence interval, *Ref* reference group

### Case-control analysis

Per the specified criteria, a match was identified for 106 (80%) of the patients receiving curative intent radiotherapy. Baseline characteristics among matched pairs: median age 71, 47% female, 71% ECOG PS 0–1, 57% weight loss < 5, and 44% current smokers. There was a higher proportion of patients with NOS histology in the radiotherapy cohort (45% vs 12%).

**Fig. 2:**
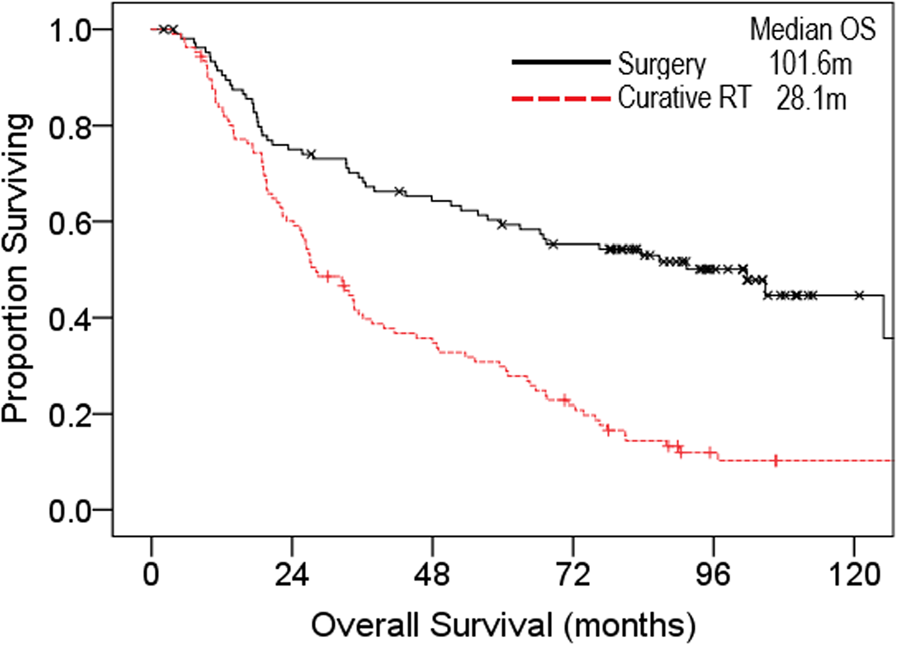
Kaplan-Meier overall survival curves in the case-control population, comparing surgery (*n* = 106) matched by age (+/− 5 years), sex, ECOG (0–1 or 2), and smoking status (never, former, current) to radiotherapy (*n* = 106) m, months; RT, radiotherapy

Median overall survival was 47.9 m in the entire matched cohort. Median OS remained superior in the surgery cohort at 101.6 m compared to 28.1 m in the CurRT cohort (Fig. 2). Five-year OS was estimated at 59% for surgery and 30% for CurRT.

### Optimizing RT

**Fig. 3:**
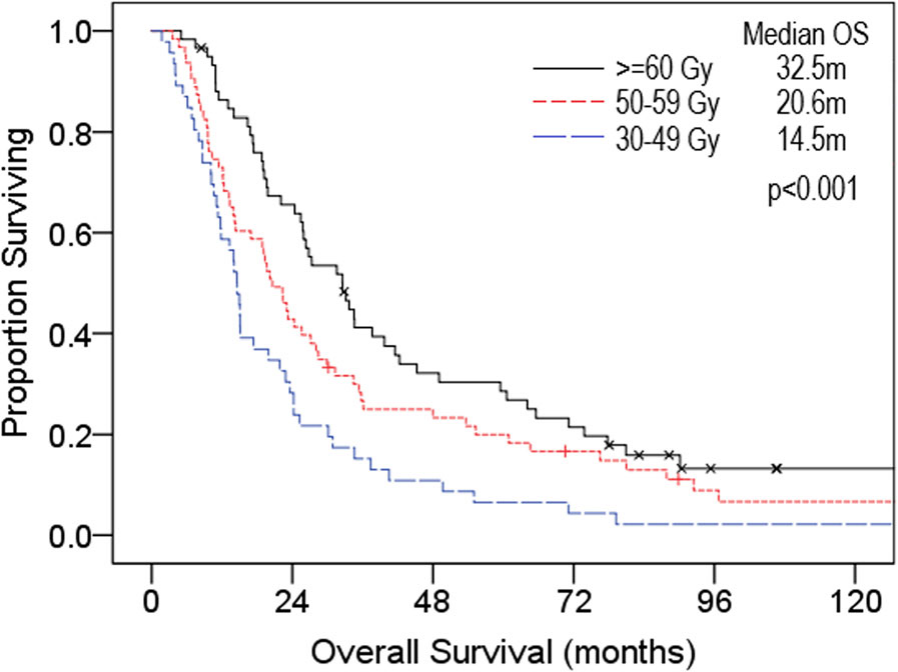
Kaplan-Meier overall survival curve in the curative RT cohort comparing concurrent/sequential chemoradiation (*n* = 34) to curative RT alone (*n* = 98) m, months; RT, radiotherapy

Within the CurRT cohort, 34 (26%) patients received concurrent or sequential chemotherapy (C/SRT), and 98 (74%) of patients received RT alone. Patients receiving C/SRT were younger (median age 66 vs 76) and had better ECOG PS (85% PS 0–1 vs 59%) than patients receiving RT alone. Median OS was longer in the C/SRT group at 33.1 m compared to 23.2 m for RT alone (Fig. 3).

**Fig. 4:**
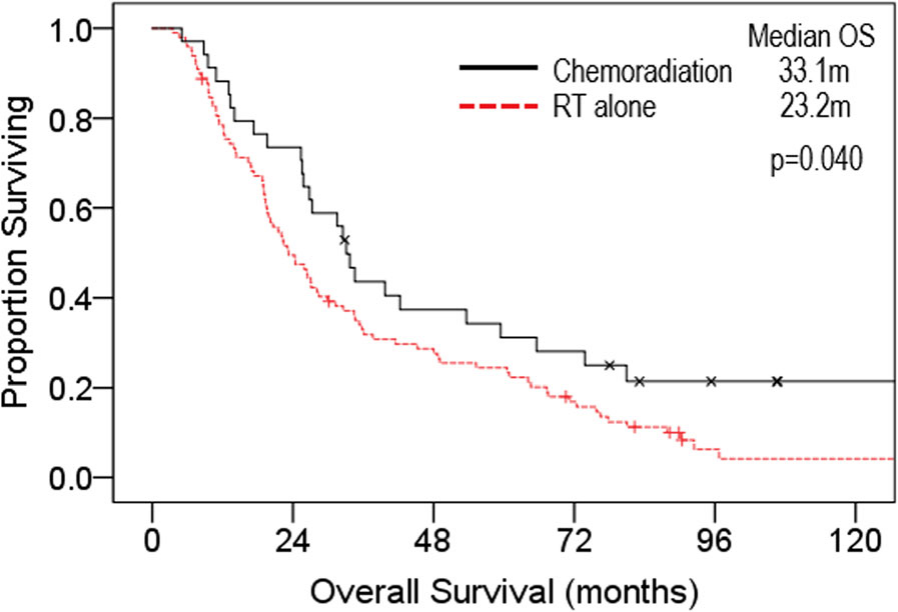
Kaplan-Meier overall survival curves of all patients treated with radiotherapy comparing > 60 Gy (*n* = 59), 50–59 Gy (*n* = 63) and high-dose palliative radiotherapy 30–49 Gy (*n* = 46)

There was a significant association between median OS and RT dose, with superior survival seen in patients receiving > = 60 Gy at 32.6 m, compared to 20.6 m for 50–59 Gy, and 14.5 m for 30–49 Gy (Fig. 4). Of the 63 patients receiving 50-59Gy, 48 (76%) received 55-59Gy, mostly at a dosing and fractionation of 55Gy/20fr. There was no significant survival difference between patients receiving 50-54Gy and 55-59Gy.

## Discussion

In a large population-based study, we evaluated the landscape of treatment and outcomes in stage II NSCLC. Although considered standard of care, less than half of patients received surgical resection. Median overall survival was 27.1 m in the entire population and differed significantly based on the type of treatment received. Surgery achieved superior survival compared to radical radiotherapy. Although this may be in part due to an imbalance in prognostic factors, the difference persisted in both a multivariate analysis and case-matched analysis.

The median overall survival in the surgery cohort was 61.4 m, and the 5-year OS was 51%. This is similar to the surgical series of the IASLC staging project for AJCC Version 7. It is sobering that despite surgical management, only half of patients are cured with the best modality available. Adjuvant chemotherapy has been shown to improve survival in stage II NSCLC [8–11], however only half of the patients in our study received adjuvant chemotherapy. This is similar to other Canadian population-based studies and may reflect a variety of factors including a lack of referral to medical oncology, patient decision, and comorbidities [20, 21]. Optimizing uptake of adjuvant chemotherapy is therefore a means to improve survival in stage II NSCLC on a population level. Exploration of alternative systemic options such as neoadjuvant therapy, immunotherapy, or targeted treatments in suitable populations are under study and may offer more patient tailored approaches for curative treatment [22–27].

A significant proportion of patients received curative intent radiotherapy due to medical inoperability or patient decision. The survival outcomes in our study were similar to previously published reports [17, 28–30], although inferior to surgery with 5-year OS being 26%. In both a multivariate model and a case-matched analysis, the performance of surgery remained superior to curative intent radiotherapy. There were a high proportion of patients in the radiotherapy cohort who died without a previously documented recurrence event. Many of these deaths are assumed to be from lung cancer, however the exact cause of death was not available. There may be a higher death rate from non-malignant causes from competing mortality due to comorbid conditions that render a patient inoperable in the first place. The radiotherapy cohort had a numerically lower number of patients with a local only recurrence, however this does not necessarily reflect better local control. Patients with distant metastases may have also had local relapse, and the high proportion of patients who died without a documented recurrence event may have died before a local relapse could develop or be confirmed.

Our findings of improved outcomes with surgery in stage II NSCLC mirror other retrospective studies in stage I disease comparing stereotactic body radiation therapy (SBRT) versus surgical resection. In a meta-analysis of over 150, 000 unmatched SBRT and surgical patients and over 17,000 matched patients, the survival was superior with surgery in both unmatched and matched populations albeit with a smaller benefit in the matched group [31]. This finding suggests that the improved outcomes must be, in part, related to differences in baseline patient and disease characteristics. In contrast, the combined analysis of prospective STARS and ROSEL studies of SBRT versus lobectomy for operable stage I NSCLC, the estimated OS at 3 years was 95% with SBRT and 79% in surgery [32]. The caveat of this analysis related to the small numbers (*n* = 58) and need to pool the data due to accrual issues. This provides some insight into the possible contribution of inoperability to outcomes when comparing groups in the retrospective studies. While these studies focus on SBRT and an earlier stage of NSCLC, it helps to highlight the contribution of baseline patient and disease characteristics to outcomes rather than the modality of therapy.

There is clear room for improvement in the outcomes for inoperable patients treated with curative intent radiotherapy. Prior studies have suggested that the addition of concurrent chemotherapy may improve survival [28], similar to what has been shown for stage III NSCLC [33]. However, the use of concurrent chemotherapy was low in our population. This may be due to both a lack of prospective evidence to support concurrent treatment, and the unique nature of the inoperable population, as many factors that make patients poor operative candidates would also make them poor candidates for chemotherapy.

Optimizing radiotherapy dose may be another means to improve outcomes in this population. Guidelines for locally advanced NSCLC suggest that a dose of 60 Gy should be used when treating with definitive radiotherapy, but it is not clear if this can be extrapolated to stage II disease [34]. Prior retrospective studies have defined curative radiotherapy for early stage NSCLC as a minimum dose of 50Gy [29, 30]. Our study showed superior outcomes in patients treated with > 60 Gy compared to those treated with lower doses of potentially curative radiotherapy, which is consistent with other retrospective studies [17]. However, doses of 50–59 Gy did portend a survival benefit over moderate-high dose palliative radiotherapy and represents a potentially curative, albeit inferior modality, with a 5-year overall survival of 21%. Most patients within this group received 55Gy/20fr, which corresponds to an equieffective dose delivered in 2 fractions (EDQ2) of 58.2Gy. Therefore, one might expect similar effectiveness to patients receiving > 60 Gy which was not seen in our study. Selection bias may play a role, as less fit patients may be selected for the shorter treatment course offered by a 55Gy/20fr dosing schedule. There was no survival difference seen between 50-54Gy and 55-59Gy, though this analysis was limited by small patient numbers.

Adjuvant immunotherapy post-chemoradiotherapy with durvalumab has shown significant improvements in survival in patients with stage III NSCLC [35, 36]. It is unknown if these benefits can be extrapolated to the population receiving radical radiotherapy or chemoradiotherapy for stage II disease. The current PACIFIC4 trial is evaluating adjuvant durvalumab after SBRT and includes node negative stage II patients (T3 N0), which may help inform this issue [37]. However, there will still be uncertainty regarding the potential role of adjuvant immunotherapy in inoperable stage II patients who are either node positive or have tumors not amenable to SBRT.

We confirmed the prognostic impact of age, histology, weight loss, and ECOG performance status. In multivariate analysis, treatment cohort and ECOG PS were the strongest predictors of survival, which is consistent with prior studies reporting ECOG PS as the most robust prognostic indicator in NSCLC [38]. There was an increase in proportion of NOS histology among the non-surgical cohorts, likely due to less tissue being available for analysis.

Although our study included patients defined as stage II disease by the AJCC 7th edition, there were only minor changes made to stage II disease in the AJCC 8th edition. Some patients included in our study, such as those with tumors greater than 7 cm, or greater than 5 cm with N1 involvement, would now be classified as having stage III disease. Therefore, the prognosis seen in our study may be slightly worse than that for a population of stage II patients defined by the AJCC 8th edition.

Our study is limited by its retrospective nature, however it is unlikely surgery and radiotherapy in this setting will ever be successfully compared in a prospective trial. In addition, comorbidity data were not available and data for known driver mutations were limited, as EGFR testing began at our institution in 2010, and ALK in 2014. The strengths include the large real-world population and the detailed information available on both baseline characteristics and treatment details. We attempted to account for the imbalance in prognostic factors between treatment cohorts in both a multivariate model and a case-matched analysis. We were able to describe the outcomes of a large group of patients treated with curative intent radiotherapy, where there is currently very limited data to guide treatment choices.

## Conclusion

There is a large proportion of patients with stage II NSCLC who are unable to receive standard of care surgical resection. Although inferior to surgery, radical radiotherapy remains a potentially curative option in inoperable patients. Optimizing radiotherapy dose and the use of concurrent chemotherapy may be strategies to improve outcomes in this population. With lung cancer screening programs leading to a migration to earlier stage at diagnosis, it will be important to focus future research efforts on this growing patient population.

## Data Availability

The datasets generated and/or analysed during the current study are not publicly available due lack of approval from Research Ethics Board, but may be available from the corresponding author on reasonable request.

## Abbreviations

AJCC: American Joint Committee on Cancer
ALK: Anaplastic lymphoma kinase
BC: British Columbia
C/SRT: Concurrent or sequential chemotherapy
CurRT: Curative intent radiation
DFS: Disease-free survival
ECOG: Eastern cooperative oncology group
EGFR: Epidermal growth factor receptor
IASLC: International association for the study of lung cancer
NOS: Not otherwise specified
NSCLC: Non-small cell lung cancer
OS: Overall survival
PS: Performance status
RT: Radiation therapy
SBRT: Stereotactic body radiation therapy
